# Complete mitochondrial genome of tiger cowrie *Cypraea tigris* (Linnaeus, 1758)

**DOI:** 10.1080/23802359.2019.1627933

**Published:** 2019-07-26

**Authors:** Liyun Pu, Hongtao Liu, Mingqiu Yang, Bingshun Li, Guangyuan Xia, Minghui Shen, Guofu Wang

**Affiliations:** Hainan Provincial Key Laboratory of Tropical Maricultural Technologies, Hainan Academy of Ocean and Fisheries Sciences, Haikou, China

**Keywords:** Mitochondrial genome, *Cypraea tigris*, phylogenetic tree

## Abstract

The complete mitochondrial genome of *Cypraea tigris* from the South China Sea has been determined, which was the first report of complete mitogenome in the superfamily Cypraeoidea. It is 16,177 bp long and consists of 21 tRNA genes, 2 rRNA genes, 13 protein-coding genes (PCGs), and 1 control region. The base composition of *C. tigris* mitogenome is biased (A, G, T, and C were 28.8, 17.9, 37.1, and 16.3%, respectively) with A + T contents of 58.5%. All of the PCGs use a typical start codon (ATN) and a complete stop codon (TAA or TAG) as normal. The maximum-likelihood tree demonstrated that the Cypraeoidea was closer to the superfamily Tonnoidea, and further clarified the phylogenetic relationships of each superfamily in Littorinimorpha.

*Cypraea tigris*, also known as the tiger cowrie or tiger cowry, is a typical large sea snail in the family Cypraeidae. It occurs in large numbers over the majority of the tropical Indo-Pacific region, from Africa to Hawaii. It is a non-aggressive, non-venomous, nocturnal, and carnivore species with an average size of 8 cm, juvenile specimens have different feeding habits than the adults and feed chiefly on algae and detritus. However, a study showed that *C. tigris* is considered reef safe with caution (Bos et al. 2011). It is one of the most well-known sea snails because of its commercially valuable shells, which not only can be collected due to a huge range of different pattern and colour variations on it, but they can be used for the production of hydroxyapatite and β-tricalcium phosphate powders by a chemical synthesis (Newton et al. 1993; Sahin et al. 2015). However, the collection by people also significantly affected its abundance (Newton et al. 1993). In addition, antimicrobial activity in tissue extracts of *C. tigris* was assayed, but no antibacterial and antifungal activities were exhibited (Anand and Edward 2002). The specimens were collected and stored in Qionghai research base of Hainan Academy of Ocean and Fisheries Sciences (19°22′21.82″ N, 110°40′37.74″ E). Muscle samples of *C. tigris* were preserved in absolute ethanol for total DNA extraction.

The complete mitochondrial genome of *C. tigris* is 16,177 bp in length (GenBank Accession No. MK783263). The total base content of *C. tigris* mitogenome was 28.8% A, 17.9% G, 37.1% T, and 16.3% C. the 58.5% of (A + T) showed a little preference to AT. It consists of 21 tRNA genes, 13 protein-coding genes (PCGs), 2 rRNA genes, and 1 control region (D-loop). Majority of the genes were encoded on heavy strand except for seven tRNA genes (*tRNA-Met*, *-Tyr*, *-Cys*, *-Trp*, *-Gly*, *-Glu*, and *tRNA-Thr*).

The mitochondrial genome of *C. tigris* contains 21 tRNA genes varying from 66 to 72 bp in length. The 12S rRNA is 972 bp and located between *tRNA-Glu* and *tRNA-Val*, and the 16S rRNA is 1388 bp, located between *tRNA-Val* and *tRNA-Leu*. For the PCGs of *C. tigris* mitogenome, all of them use the typical initiation codon ATN (10 genes use ATG; *COX3* and *ND4L* use ATA; *ND4* uses ATT); all of them end with a complete stop codon (9 genes use TAA; 4 genes use TAG). The control region located between *tRNA-Phe* and *COX3* with a length of 911 bp. Interestingly, in this region, (AT)_11_ microsatellites (SSRs) were identified using MISA.

The complete mitogenome of *C. tigris* was the first report of complete mitogenome in the superfamily Cypraeoidea. A phylogenetic analysis was compiled based on 13 PCGs genes encoded by 20 Littorinimorpha mitogenomes available in GenBank using the maximum-likelihood (ML) method with 1000 bootstrap replicates. The phylogenetic tree (Figure 1) demonstrated that the Cypraeoidea was closer to the superfamily Tonnoidea, and further clarified the phylogenetic relationships of the superfamily in Littorinimorpha compared with the previous work by single mitochondrial genes (Meyer 2003; Sun et al. 2012).

**Figure 1.**
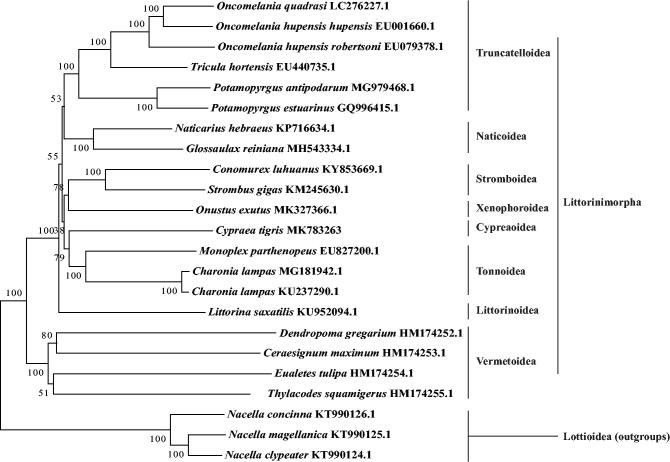
A maximum-likelihood tree illustrates the phylogenetic position of *C. tigris* among other species.
